# Biomarkers in Cardiorenal Syndrome and Potential Insights Into Novel Therapeutics

**DOI:** 10.3389/fcvm.2022.868658

**Published:** 2022-05-20

**Authors:** Edmund Y. M. Chung, Katie Trinh, Jennifer Li, Sebastian Hayden Hahn, Zoltan H. Endre, Natasha M. Rogers, Stephen I. Alexander

**Affiliations:** ^1^Centre for Kidney Research, The Children’s Hospital at Westmead, Westmead, NSW, Australia; ^2^Centre for Transplant and Renal Research, Westmead Institute for Medical Research, Westmead, NSW, Australia; ^3^Faculty of Science, University of New South Wales, Kensington, NSW, Australia; ^4^Department of Nephrology, Prince of Wales Hospital, Randwick, NSW, Australia; ^5^Faculty of Medicine, University of New South Wales, Kensington, NSW, Australia; ^6^Department of Renal Medicine, Westmead Hospital, Westmead, NSW, Australia; ^7^Department of Nephrology, The Children’s Hospital at Westmead, Westmead, NSW, Australia

**Keywords:** cardiorenal syndrome (CRS), biomarker (BM), heart failure, chronic kidney disease, prognosis

## Abstract

Heart and kidney failure often co-exist and confer high morbidity and mortality. The complex bi-directional nature of heart and kidney dysfunction is referred to as cardiorenal syndrome, and can be induced by acute or chronic dysfunction of either organ or secondary to systemic diseases. The five clinical subtypes of cardiorenal syndrome are categorized by the perceived primary precipitant of organ injury but lack precision. Traditional biomarkers such as serum creatinine are also limited in their ability to provide an early and accurate diagnosis of cardiorenal syndrome. Novel biomarkers have the potential to assist in the diagnosis of cardiorenal syndrome and guide treatment by evaluating the relative roles of implicated pathophysiological pathways such as hemodynamic dysfunction, neurohormonal activation, endothelial dysfunction, inflammation and oxidative stress, and fibrosis. In this review, we assess the utility of biomarkers that correlate with kidney and cardiac (dys)function, inflammation/oxidative stress, fibrosis, and cell cycle arrest, as well as emerging novel biomarkers (thrombospondin-1/CD47, glycocalyx and interleukin-1β) that may provide prediction and prognostication of cardiorenal syndrome, and guide potential development of targeted therapeutics.

## Introduction

Heart failure (HF) and chronic kidney disease (CKD) are increasing public health issues with a prevalence of 4% and 9% respectively, and a global rise in attributable deaths by 41% for both diseases since 1990 (1, 2). Cardiorenal syndrome (CRS) refers to the concurrent dysfunction of the heart and kidney, which can initiate and perpetuate disease in the other organ through hemodynamic, neurohormonal, and immunological and/or biochemical feedback pathways (3). Combined HF and CKD is associated with high morbidity and mortality (4–9). In people with HF, every 10 ml/min decrease in estimated glomerular filtration rate (eGFR) increases the risk of all-cause death by 7%, while HF hospitalization in people with CKD increases the risk of all-cause death 3–7-fold (8, 9).

## Classification of Cardiorenal Syndrome

Currently, CRS is classified into 5 different subtypes based on the perceived primary precipitant of organ injury: (10)

1. CRS type 1: rapid decline in cardiac function (e.g., cardiogenic shock or acute decompensation of chronic HF) resulting in acute kidney injury (AKI).
2. CRS type 2: chronic cardiac dysfunction (e.g., chronic HF) causing progressive decline in kidney function and CKD.
3. CRS type 3: acute decline in kidney function (e.g., AKI or glomerulonephritis) causing acute cardiac dysfunction (e.g., HF, arrhythmia, or myocardial infarction).
4. CRS type 4: CKD causing progressive decline in cardiac function (e.g., left ventricular hypertrophy, HF, or myocardial infarction).
5. CRS type 5: combine cardiac and kidney dysfunction caused by an acute or chronic systemic disorder (e.g., sepsis or diabetes mellitus).

While this classification system is clinical intuitive, it may be difficult to identify the initial insult. Furthermore, this classification system does not incorporate the pathophysiological pathways implicated in CRS such as hemodynamic dysfunction, neurohormonal activation, endothelial dysfunction, inflammation, and fibrosis (11, 12).

**FIGURE 1:**
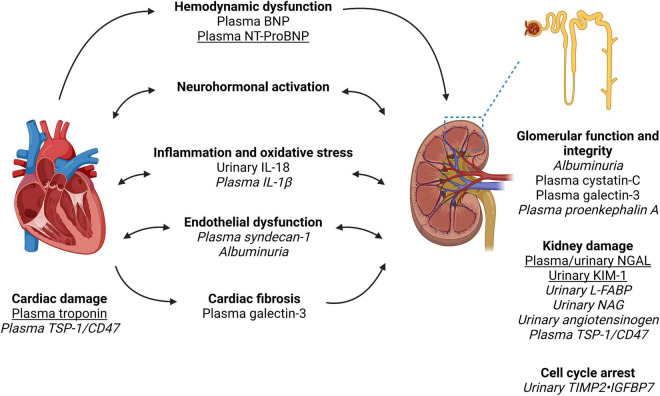
Biomarkers in the pathways of injury in cardiorenal syndrome. Created with Biorender. Cardiorenal syndrome involves the complex bidirectional nature of heart and kidney dysfunction. The pathways involved include hemodynamic dysfunction, neurohormonal activation (primarily the renin-angiotensin-aldosterone system), inflammation and oxidative stress, endothelial dysfunction, and ultimately injury and fibrosis to both heart and kidney. We have outlined the pathways which each biomarker is associated with as well as indicated biomarkers with the most clinical applicability based on the evidence quality of existing literature (underline) vs. biomarkers requiring further research and validation in larger cohort of patients with cardiorenal syndrome (italics). Of note, biomarker performance will depend on kidney function, timing of collection and type of cardiorenal syndrome. Furthermore, a combination of biomarkers may have superior performance to a single biomarker. Therefore, further research is required to identify the type and timing of biomarker(s) analysis in the prediction and prognostication of cardiorenal syndrome. NGAL, neutrophil gelatinase-associated lipocalin; KIM-1, kidney injury molecule-1; L-FABP, liver fatty acid-binding protein; NAG, *N*-acetyl-beta-D-glucosaminidase; BNP, brain natriuretic peptide; NT-proBNP, N-terminal pro-brain natriuretic peptide; IL, interleukin; TIMP2, tissue inhibitor of metalloproteinases-1 and -2; IGFBP7, insulin-like growth factor-binding protein-7; TSP-1, thrombospondin-1.

Current biomarkers of kidney function such as serum creatinine also have limited sensitivity and specificity. A rise in serum creatinine only occurs after a significant decline in GFR (e.g., 48–72 h after AKI or after 50% of function is lost chronically), is non-specific to the underlying disease process, and is affected by clinical characteristics (e.g., age, weight, gender, ethnicity, volume status, and medication use) that do not reflect true parenchymal injury (13). In this review, we summarize the evidence for different biomarkers in CRS (Figure 1 and Table 1) as well as promising emerging biomarkers that may inform on future management in this debilitating condition.

**TABLE 1:** Biomarkers in cardiorenal syndrome.

Biomarker	Function of biomarker	Predictive value (AUC-ROC per outcome)	Prognostic value (“x” times increased risk of outcome)	Involved in the disease mechanism of CRS	Targeted treatments	References
**Biomarkers of function (glomerular filtration and integrity)**						
Albuminuria	Marker of glomerular injury	Unclear	Type 2 CRS: -All-cause/CV death or HF hospitalization: 1.4–1.8 times	No	RAAS inhibitors, MRAs, SGLT2 inhibitors	15–17
Plasma cystatin-C	Produced by all nucleated cells. Marker of GFR	Type 1 CRS: -AKI: 0.68 -All-cause death or hospitalization: 0.73	Type 1 and 2 CRS: -All-cause death: 2–3 times	No	No	19–28
Plasma galectin-3	Involved in RAAS-mediated collagen deposition by fibroblasts. Also inversely related to GFR	Acute HF: -All-cause death: 0.74 Chronic HF: -All-cause death: 0.612 -All-cause death or HF hospitalization: 0.58	Type 1 CRS: -All-cause death or HF hospitalization: 14 times Type 2 CRS: -All-cause death or HF hospitalization: 1.6–2 times	Yes	RAAS inhibitors, MRAs	30–33
Plasma proenkephalin A	Involved in opioid receptor-mediated negative inotropic effects. Also inversely related to GFR	Type 1 CRS: -AKI: 0.69	Type 1 CRS: -All-cause death or HF hospitalization: 1.3 times	No	No	35
**Biomarkers of kidney damage**						
Plasma and/or urinary NGAL	Secreted by neutrophils and epithelial cells in response to inflammation. Mediates cardiac fibrosis by aldosterone	Type 1 CRS: -AKI: 0.775–0.998	Type 1 CRS: -All-cause death: 1.3–2 times -AKI: 5 times	Yes	No	22–23, 38–41, 44–46
Urinary KIM-1	Facilitates phagocytosis of apoptotic renal tubular cells	Type 1 CRS: -AKI: 0.83–0.88	Type 1 CRS: -All-cause death: 2 times Type 2 CRS -All-cause death or HF hospitalization: 1.1–1.5 times	No	No	22, 47–52
Urinary IL-18	Marker of injury from NLRP3-inflammasome on cardiac myocytes and renal tubular cells	Type 1 CRS: -AKI: 0.61–0.75 -AKI-to-CKD: 0.674	Type 1 CRS: -AKI: 3.6 times -All-cause death: 1.2 times	No	No	46–47, 52, 56
Urinary L-FABP	Binds fatty acid oxidation products	Type 1 CRS: -AKI: 0.86 when urinary L-FABP/NAG combined	Unclear	No	No	58
Urinary NAG	Renal proximal tubule brush border marker		Type 2 CRS: -All-cause death: 1.3–1.4 times -HF hospitalization: 1.2 times	No	No	48–49, 58
Urinary angiotensinogen	Marker of intrarenal RAAS activation	Type 1 CRS: -AKI: 0.78 -All-cause death: 0.85	Unclear	Yes	RAAS inhibitors, MRAs	46
**Biomarkers of cardiac damage**						
Plasma cTnT	Marker of cardiac myocyte injury	Type 4 CRS: -AMI: sensitivity 92–95%, specificity 88–97% *	Type 4 CRS: -CV events: 2–6 times -All-cause death: not associated	No	No	60–64
Plasma BNP and NT-proBNP	Marker of left ventricular wall stretch	Type 4 CRS: -All-cause death: 0.699–0.818 -All-cause death or CV events: 0.666–0.720?	Type 4 CRS: -CV events: 1.4 times -All-cause death: 1.6 times	Yes	Diuretics	60, 66–67
**Cell cycle arrest biomarkers**						
Urinary [TIMP2]•[IGFBP7]	Involved in G1 cell-cycle arrest during early phases of cell injury	Type 1 CRS: -AKI: 0.75–0.84	Unclear	No	No	71–72
**Novel biomarkers**						
Plasma TSP-1	Binds to CD47 to limit nitric oxide-mediated vasodilation	AMI: -HF: 0.82	Unclear	Yes	Anti-CD47 blockade and microRNA-221 targeting TSP-1 in animal models	84, 86–88
Plasma syndecan-1	Marker of glycocalyx injury	Type 1 CRS: -AKI: 0.741 -Severe AKI: 0.812 -All-cause death: 0.788	Type 1 CRS: -All-cause death: 1.3 times Chronic HF: -All-cause death or hospitalization: 2 times (HFpEF) -Not prognostic for HFrEF	Yes	Glycocalyx-protective treatments (albumin, sulodexide, FFP, steroids, etanercept, statins, metformin, heparin)	94–95, 97–111
Plasma IL-1β	Marker of injury from NLRP3-inflammasome on cardiac myocytes and renal tubular cells	Unclear	Unclear	Yes	Anti-IL-1β blockade (canakinumab) in human CV disease	113

*CRS, cardiorenal syndrome; AUC-ROC, area under the receiver operating characteristic curve; AKI, acute kidney injury; CV, cardiovascular; HF, heart failure; RAAS, renin-angiotensin-aldosterone system; MRA, mineralocorticoid receptor antagonists; SGLT2, sodium-glucose co-transporter 2; GFR, glomerular filtration rate; NGAL, neutrophil gelatinase-associated lipocalin; KIM-1, kidney injury molecule-1; L-FABP, liver fatty acid-binding protein; NAG, N-acetyl-beta-D-glucosaminidase; cTnT, cardiac troponin T; AMI, acute myocardial infarction; CKD, chronic kidney disease; BNP, brain natriuretic peptide; NT-proBNP, N-terminal pro-brain natriuretic peptide; IL, interleukin; GDF-15, growth differentiation factor 15; ST2, suppressor of tumorigenicity 2; TIMP2, tissue inhibitor of metalloproteinases-1 and -2; IGFBP7, insulin-like growth factor-binding protein-7; TSP-1, thrombospondin-1; HFpEF, heart failure with preserved ejection fraction; HFrEF, heart failure with reduced ejection fraction; FFP, fresh frozen plasma.*

*cTnT cut-off > 43.2 ng/L for pre-dialysis CKD, > 350 ng/L for kidney failure.

*Cut-offs differ for all-cause death (stage 1–3 CKD: BNP > 90.8 pg/ml and NT-proBNP > 259.7 pg/ml and stage 4–5 CKD: NT-proBNP > 2,584.1 pg/mL) and all-cause death or HF hospitalization (stage 4–5 CKD: BNP > 157.0 pg/ml and NT-proBNP > 5,111.5 pg/ml).*

## Biomarkers of Function (Glomerular Filtration and Integrity)

### Albuminuria

Albuminuria is a cheap and widely available biomarker, which may not only reflect glomerular injury but also endothelial dysfunction (14). In three large chronic HF trials, microalbuminuria (30–299 mg/g) and macroalbuminuria (≥ 300 mg/g) were associated with a 1.4–1.8-fold increased risk of all-cause death, cardiovascular death, or HF hospitalization (15–17). Albuminuria predicted these outcomes independent of serum creatinine, HbA1c and left ventricular ejection fraction (LVEF) (16). While treatments exist to reduce albuminuria [e.g., renin-angiotensin-aldosterone system (RAAS) inhibitors], whether targeting albuminuria improves prognosis in CRS requires further study.

### Cystatin-C

Cystatin-C, a 13-kDa cysteine protease inhibitor, is produced at a constant rate by all nucleated cells and is freely filtered through the glomerulus, almost completely reabsorbed, and not secreted by the renal tubules. Cystatin-C inhibits collagen- and elastin-degrading cysteine proteases of the cathepsin family and protects against atherosclerosis in apolipoprotein E–deficient mice, though its role in CRS is unclear (18). In both acute and chronic HF, elevated plasma or urinary cystatin-C was associated with a 2–3-fold increased risk of all-cause death, independent of serum creatinine or eGFR (19–22). In acute HF, plasma cystatin-C modestly predicted AKI [area under the receiver operating characteristic curve (AUC-ROC) 0.68] and all-cause death or HF hospitalization (AUC-ROC, 95% confidence interval: 0.73, 0.66–0.80) (23, 24), providing prognostic value in addition to N-terminal prohormone of brain natriuretic peptide (NT-proBNP) and troponin (25). In elderly patients with chronic HF, the highest quartile of cystatin-C doubled the risk of all-cause death, outperforming serum creatinine in multivariate analyses (21). After cardiac surgery, plasma cystatin-C modestly predicted AKI (AUC-ROC 0.68), (26) while urinary cystatin-C has not consistently predicted AKI (27, 28).

Unlike serum creatinine, plasma cystatin-C is not affected by muscle mass but both are affected by volume status. In the Renal Optimization Strategies Evaluation–Acute Heart Failure (ROSE-HF) trial, protocol-driven aggressive diuresis caused worsening of renal function (WRF), based on ≥ 20% decrease in eGFR using cystatin C, in 21% of participants but was not associated with an increase in kidney tubular injury markers (29). This suggests tubular markers may have utility in differentiating AKI due to diuretic-induced volume depletion or parenchymal injury in CRS.

### Galectin-3

Galectin-3 is a 30-kDa glycoprotein synthesized by cardiac macrophages in response to angiotensin II and aldosterone, which mediates collagen deposition by fibroblasts resulting in cardiac fibrosis (12). Plasma galectin-3 levels are also inversely related to renal function and therefore represents a biomarker for both cardiac fibrosis and GFR. In acute HF, while NT-proBNP was superior to galectin-3 for diagnosis, galectin-3 may be superior to NT-proBNP at predicting 60-day mortality (AUC-ROC 0.74 vs. 0.67, *p* = 0.05) and was associated with a 14-fold increased risk of all-cause death or HF hospitalization in multivariate analysis (30). In chronic HF, galectin-3 only modestly predicted all-cause death (AUC-ROC 0.612, 0.538–0.685) and all-cause death or HF hospitalization (AUC-ROC 0.58, 0.55–0.61). Pooled analysis of chronic HF trials (mean eGFR 54–58 ml/min/1.73 m^2^) showed elevated galectin-3 was associated with a 1.6–2-fold increased risk of all-cause death or HF hospitalization (31–33).

### Proenkephalin A

Proenkephalin A is an endogenous opioid secreted by cardiac cells and mediates negative inotropic effects *via* the delta opioid receptor. Plasma proenkephalin is inversely proportional to GFR and associated with a 1.5-fold increased risk of CKD (highest vs lowest tertile) (34). In acute HF, proenkephalin A modestly predicted AKI (AUC-ROC 0.69) and was independently associated with a 27% increased risk of 1-year mortality or HF hospitalization (35).

## Biomarkers of Damage

### Kidney Tubule: Neutrophil Gelatinase-Associated Lipocalin

Neutrophil gelatinase-associated lipocalin (NGAL) is a 25-kDa protein secreted by immature neutrophils, epithelial cells (including renal tubular epithelium) and cardiomyocytes in response to inflammation. While NGAL has been extensively studied as a marker of renal tubular injury, it also plays a role in mineralocorticoid-mediated cardiovascular fibrosis. NGAL knockout mice demonstrated blunted vascular fibrosis in response to an aldosterone-salt challenge, and reduced cardiac fibrosis, inflammation and left ventricular dysfunction in a myocardial infarction model (36, 37). However, no treatments exist to inhibit NGAL for evaluation in CRS.

In neonatal and pediatric cardiac surgery, plasma and urinary NGAL were detectable 2 h post-surgery (compared to 1–3 days for serum creatinine) and performed exceptionally at predicting AKI (AUC-ROC 0.92–0.998) (38, 39). A small study in adult cardiac surgery demonstrated similar predictive ability for detecting AKI (AUC-ROC 0.98), (40) but this has not been consistent replicated. In a meta-analysis of NGAL in diagnosing AKI, subgroup analysis of 10 studies reporting cardiac surgery-related AKI demonstrated plasma and/or urinary NGAL moderately predicted AKI (AUC-ROC 0.775, 0.669–0.867) (41). In a large cohort study of adults undergoing cardiac surgery, plasma (but not urinary) NGAL significantly improved risk prediction of AKI over the clinical models using demographic factors, surgical factors, eGFR and patient comorbidities (42). These differences highlight the importance of considering time and baseline kidney function when assessing biomarkers. Indeed, for the diagnosis of AKI in 529 critically ill patients in the intensive care unit, cystatin-C, interleukin-18 (IL-18), NGAL, kidney injury molecule-1 (KIM-1), and γ-glutamyltranspeptidase demonstrated optimal performance earlier (≤ 12 h after injury) in patients with preserved kidney function (eGFR ≥ 60 ml/min) and later (12–36 h after injury) in patients with reduced kidney function (eGFR < 60 ml/min) (43). In acute HF, plasma and urinary NGAL were associated with a 1.3–2-fold increased risk of long-term mortality, (22, 44) and plasma NGAL outperformed cystatin-C at predicting AKI (AUC-ROC 0.93 vs. 0.68) (23). Plasma NGAL kinetics may also improve its ability to predict AKI (AUC-ROC 0.91 for delta NGAL change vs. 0.69 for NGAL at baseline) (45). Lastly, urinary NGAL performed 2 days after hospitalization for acute HF differentiated true WRF from pseudo-WRF based on AKI with or without clinical improvement (AUC-ROC 0.83, 0.73–0.93), (22) was associated with a 5-fold risk of AKI, (46) but failed to predict AKI-to-CKD transition (47).

### Kidney Tubule: Kidney Injury Molecule-1

Kidney injury molecule-1 (KIM-1) is a transmembrane glycoprotein with immunoglobulin and mucin domains, which is expressed by renal proximal tubule epithelium in response to injury and facilitates phagocytosis of apoptotic tubular cells. In chronic HF, elevated urinary KIM-1 was modestly associated with a 10–15% increased risk of all-cause death or HF hospitalization but did not predict AKI nor AKI-to-CKD transition in acute HF (22, 47–49). In contrast, urinary KIM-1 measured 12 h after cardiac surgery predicted AKI with good performance (AUC-ROC 0.83–0.88), (50, 51) and a high cut-off for urinary KIM-1 (highest vs. lowest tertile) was associated with a doubling of 3-year mortality risk, independent of post-operative AKI (52).

### Kidney Tubule: IL-18

Interleukin-18 (IL-18) is an 18-kDa pro-inflammatory cytokine produced by immune cells (e.g., macrophages) and non-immune cells (e.g., vascular endothelial cells and renal proximal tubular cells) in response to tissue injury *via* activation of the NLRP3-inflammasome, which causes programmed cell death in both cardiomyocytes and renal tubular cells (53–55). In acute HF, elevated urinary IL-18 was associated with a 3.6-fold increased risk of AKI in multivariate analysis, (46) and modestly predicted AKI-to-CKD transition at 6 months (AUC-ROC 0.674, 0.543-0.805) (47). In people undergoing cardiac surgery, urinary IL-18 also modestly predicted AKI (AUC-ROC 0.61 at 4 h post-surgery, 0.75 at 12 h, and 0.73 at 24 h), (56) which was superior to clinical models using eGFR, (42) and was associated with a modest 1.2-fold increased risk of long-term mortality (52).

### Kidney Tubule: Liver Fatty Acid-Binding Protein (L-FABP), *N*-Acetyl-Beta-D-Glucosaminidase (NAG) and Urinary Angiotensinogen

Fatty-acid binding proteins (FABPs) are a family of 15-kDa cytoplasmic proteins that are involved in the intracellular transport of long-chain fatty acids. L-FABP is located on the renal proximal tubular cells may reduce oxidative stress by binding fatty acid oxidation products (57). NAG is a large lysosomal brush border enzyme (>130 kDa), predominantly expressed on proximal tubular cells, and is not freely filtered. In patients undergoing cardiac surgery, combining urinary L-FABP and NAG at 4 h after surgery with pre-operative clinical factors (including demographics, comorbidities and serum creatinine) improved AKI prediction compared to pre-operative clinical factors alone (AUC-ROC 0.86, 0.74–0.93 vs. 0.79, 0.66–0.88, *p* < 0.05) (58). In chronic HF, elevated urinary NAG was also associated with a 1.2-fold increased risk of HF hospitalization and 1.3–1.4-fold increased risk of all-cause death in multivariate models (48, 49). In acute HF, urinary angiotensinogen also predicted AKI (AUC-ROC 0.78) and all-cause death (AUC-ROC 0.85) (46).

### Cardiac Myocyte: Troponin

Cardiac troponin I (cTnI) and T (cTnT) are established diagnostic and prognostic biomarkers in myocardial infarction and HF (59). While troponin levels are elevated in people with CKD due to reduced plasma clearance, elevated cTNT was associated with a 2–6-fold increased risk of cardiovascular events, (60, 61) but not all-cause death after adjusting for kidney function (62). Adjusting cTnT cut-off retained good performance at detecting acute myocardial infarction in people with CKD (cTnT > 350 ng/L (standard cut-off > 14 ng/L) for eGFR < 15 ml/min/1.73 m^2^: sensitivity 95%, specificity 97%; cTnT cut-off > 43.2 ng/L for eGFR < 60 ml/min/1.73 m^2^: sensitivity 92%, specificity: 88%) (63, 64).

### Cardiac Ventricular Stretch: Brain Natriuretic Peptide and N-Terminal proBNP

Pro-BNP is secreted by cardiomyocytes in the ventricle and atria in response to ventricular wall stretch and cleaved into active BNP and inactive NT-proBNP, which are both established biomarkers in HF (65). While NT-proBNP is elevated in people with CKD, elevated NT-proBNP was still associated with a 1.3-fold increased risk of cardiovascular events and 1.6-fold increased risk of all-cause death after adjusting for kidney function (60). In people with CKD, both BNP and NT-proBNP demonstrated moderate predictive ability for all-cause death and/or cardiovascular events at different cut-offs. For stage 1–3 CKD, BNP > 90.8 pg/ml (AUC-ROC 0.699) and NT-proBNP > 259.7 pg/ml (AUC-ROC 0.702) predicted all-cause death. In comparison, for stage 4–5 CKD, BNP > 157.0 pg/ml (AUC-ROC 0.666) and NT-proBNP > 5,111.5 pg/ml (AUC-ROC 0.720) predicted all-cause death or cardiovascular events (66). NT-proBNP kinetics may have improved clinical utility in the CKD population with a doubling of NT-proBNP associated with a 1.4-fold increased risk of cardiovascular events in African-Americans with CKD in multivariate analysis (67).

## Biomarkers of Cell Cycle Arrest

### Tissue Inhibitor of Metalloproteinase-2 x Insulin-Like Growth Factor-Binding Protein-7 [TIMP2]•[IGFBP7]

TIMP2 and IGFBP7 are involved in G1 cell-cycle arrest during early phases of cell injury and the product of urinary TIMPIGFBP7 concentrations (NephroCheck) is the first Food and Drug Administration-approved test to assess the risk of AKI based on studies in critically ill patients in the intensive care unit (68–70). Small studies have demonstrated urinary [TIMP2]•[IGFBP7] predict AKI after cardiac surgery (AUC-ROC 0.84), (71) and in acute HF, where it outperformed KIM-1 (AUC-ROC 0.75, 0.61–0.88 vs. 0.54, 0.37–0.70) (72).

## Current Challenges in the Implementation of Biomarkers and Emerging Novel Biomarkers

The forementioned biomarkers demonstrate diagnostic utility for AKI and/or cardiac events though implementation remains challenging. Key suggestions for biomarker use from the 23rd Acute Disease Quality Initiative meeting included combining damage and functional biomarkers to identify patients at high-risk of AKI, to improve the diagnostic accuracy of AKI, discriminate AKI etiology, and to assess AKI severity (73). In CRS, biomarkers may assist our understanding of the interaction of heart-kidney injury. Kidney damage biomarkers (e.g., plasma/urinary NGAL, urinary IL-18, urinary KIM-1, urinary L-FABP, urinary NAG and urinary angiotensinogen) and cell cycle arrest biomarkers (e.g., urinary [TIMP2]•[IGFBP7]) may inform in CRS type 1, and cardiac damage biomarkers (e.g., troponin and BNP) in CRS type 3 and 4. In contrast, the utility of biomarkers in CRS type 2 and 5 require further study though potentially more sensitive markers of GFR (e.g., plasma cystatin-C and proenkephalin A) may be inform in CRS type 2, plasma galectin-3 may inform in both CRS type 2 and 4 since it is both a marker of GFR and cardiac fibrosis, and CRS type 5 likely requires a combination of biomarkers since it reflects simultaneous heart and kidney injury. However, interventions in CRS remain limited and few existing biomarkers (e.g., albuminuria, plasma galectin-3, urinary angiotensinogen, plasma BNP and NT-proBNP) offer insights into therapeutic targets that may benefit patients with CRS.

Loop diuretics remain the first-line treatment for fluid removal in HF, however, animal studies raise concerns regarding frusemide-mediated RAAS activation and subsequent myocardial and renal fibrosis (3). While aldosterone-mediated fibrosis in CRS suggest RAAS inhibitors and mineralocorticoid receptor antagonists may be beneficial, the risk of hyperkalemia or AKI may limit their use (74). The thrombospondin-1/CD47 axis, glycocalyx and IL-1β are emerging biomarkers in cardiovascular disease which may also provide prognostic value and therapeutic targets in CRS.

### Thrombospondin-1 (TSP-1)/CD47 Axis

TSP-1 is a 480 kDa matricellular protein secreted by tissue in response to hypoxia and binds to the ubiquitously expressed CD47 to limit nitric oxide (NO)-mediated vasodilation, thereby limiting tissue perfusion (75, 76). The TSP-1/CD47 axis has been implicated in renal ischemia reperfusion injury (IRI), (77, 78) atherosclerosis, (79) endothelial dysfunction, (80, 81) pulmonary hypertension, (82) and vaso-occlusive events in sickle cell anemia (83). In microarray analysis of peripheral blood samples, TSP-1 outperformed BNP and cTnT at predicting HF after myocardial infarction (AUC-ROC 0.82 vs. 0.63), (84) and plasma TSP-1 levels are also increased in people with CKD (85).

In human hearts after autopsy and experimental myocardial infarction in mice, CD47 is upregulated on cardiomyocytes and inhibited phagocytosis of apoptotic cardiomyocytes by macrophages (86). CD47 inhibition ameliorated myocardial infarction in mice and rats by enhancing myocardial phagocytosis, resolving monocyte infiltration, increasing endothelial nitric oxide synthase activity and reducing oxidative stress, resulting in reduced infarct size, reduced cardiac fibrosis, and improved left ventricular ejection fraction (86, 87). Anti-CD47 blockade also successfully ameliorated kidney fibrosis in a renal IRI model and reduced expression of fibrosis markers such as transforming growth factor (TGF)-β, SMAD2 and connective tissue growth factor (85). More relevant to CRS, microRNA-221 inhibits TSP-1 upregulation, reduces TGF-β1-mediated cardiac fibrosis, and improves cardiac function and survival in 5/6 nephrectomy rats, a model of CKD (88). Overall, the TSP-1/CD47 axis represents a promising biomarker and therapeutic target in CRS.

### Syndecan-1

The glycocalyx is a 0.5–8 μm thick carbohydrate-rich structure composed of glycoproteins (e.g., syndecan-1) bound to glycosaminoglycan side-chains (e.g., heparan sulfate and hyaluronan), which overlies vascular endothelial cells and governs its barrier function as well as antiadhesive and anticoagulant properties (89, 90). Degradation of the glycocalyx has been proposed as an early marker of endothelial dysfunction, which has been increasingly recognized as a critical process in CRS (11, 91–93). In acute HF, elevated serum syndecan-1 at hospital admission predicted AKI (AUC-ROC 0.741), severe AKI (AUC-ROC 0.812) and in-hospital death (AUC-ROC 0.788), and was associated with a 1.3-fold increased risk of all-cause death at 6 months in multivariate analysis (94). In chronic HF, elevated serum syndecan-1 was prognostic in HF with preserved ejection fraction (2-fold increased risk of all-cause death or hospitalization) but not HF with reduced ejection fraction (95).

Restoration of the glycocalyx using a novel selectin-targeting glycocalyx mimetic (DS-IkL) reduced selectin-mediated neutrophil and macrophage infiltration, endothelial cell and fibroblast proliferation, and cardiac fibrosis after myocardial infarction in mice (96). Therapies with proven safety in humans repurposed for glycocalyx regeneration (e.g., albumin, sulodexide, fresh frozen plasma, hydrocortisone, etanercept, rosuvastatin, metformin, and heparin) may also represent potential novel treatments in CRS (97–111).

### Interleukin-1β (IL-1β)

Similar to IL-18, IL-1β is involved in activation of the NLRP3-inflammasome in myocardial and kidney injury. Rademaker et al. recently performed RNA sequencing on serial kidney biopsies in an ovine model of acute HF and identified 675 differentially expressed genes with human homologs that were enriched for 9 pathways, of which IL-1β was the top-predicted upstream regulator gene (112). Canakinumab, a humanized monoclonal antibody targeting IL-1β, reduced cardiovascular events by 15% in 10,061 patients with previous myocardial infarction though at the cost of increased fatal infections (113). Similar results were reported in the CKD subgroup (114). Whether canakinumab or other biomarkers identified in the study by Rademaker et al. have a role in the treatment of CRS require further study.

## Conclusion

Cardiorenal syndrome is an increasingly common condition in the aging multimorbid population with significant health burden and few effective treatments. Current studies of biomarkers in CRS have largely focused on prognostication and clinical translation has been limited by sparse data comparing them to traditional biomarkers such as serum creatinine. Furthermore, few biomarkers offer insights into the mechanistic basis of disease needed to inform therapeutic strategies. In this review, we propose the TSP-1/CD47 axis, glycocalyx and IL-1β as promising areas for future research in CRS, which have the potential to prognosticate and direct treatments in this complex condition.

## Author Contributions

EC, NR, and SA conceptualized review topic. EC, KT, JL, SH, ZE, NR, and SA contributed to data interpretation. ZE, NR, and SA contributed to supervision. All authors contributed to important intellectual content during manuscript drafting or revision and agrees to be personally accountable for the individual’s own contributions and to ensure that questions pertaining to the accuracy or integrity of any portion of the work, even one in which the author was not directly involved, are appropriately investigated and resolved, including with documentation in the literature if appropriate.

## Conflict of Interest

The authors declare that the research was conducted in the absence of any commercial or financial relationships that could be construed as a potential conflict of interest. The handling editor YL declared a past collaboration with the author EC.

## Publisher’s Note

All claims expressed in this article are solely those of the authors and do not necessarily represent those of their affiliated organizations, or those of the publisher, the editors and the reviewers. Any product that may be evaluated in this article, or claim that may be made by its manufacturer, is not guaranteed or endorsed by the publisher.
